# Melatonin in Hypoxic-Ischemic Brain Injury in Term and Preterm Babies

**DOI:** 10.1155/2019/9626715

**Published:** 2019-02-20

**Authors:** Justyna Paprocka, Marek Kijonka, Beata Rzepka, Maria Sokół

**Affiliations:** ^1^Department of Pediatric Neurology, School of Medicine in Katowice, Medical University of Silesia, Katowice, Poland; ^2^Department of Medical Physics, Maria Skłodowska-Curie Memorial Cancer Center and Institute of Oncology Gliwice Branch, Poland; ^3^Students' Scientific Society, Department Pediatric Neurology, School of Medicine in Katowice, Medical University of Silesia, Katowice, Poland

## Abstract

Melatonin may serve as a potential therapeutic free radical scavenger and broad-spectrum antioxidant. It shows neuroprotective properties against hypoxic-ischemic brain injury in animal models. The authors review the studies focusing on the neuroprotective potential of melatonin and its possibility of treatment after perinatal asphyxia. Melatonin efficacy, low toxicity, and ability to readily cross through the blood-brain barrier make it a promising molecule. A very interesting thing is the difference between the half-life of melatonin in preterm neonates (15 hours) and adults (45-60 minutes). Probably, the use of synergic strategies—hypothermia coupled with melatonin treatment—may be promising in improving antioxidant action. The authors discuss and try to summarize the evidence surrounding the use of melatonin in hypoxic-ischemic events in term and preterm babies.

## 1. Introduction

Melatonin acting as a direct scavenger is able to remove singlet oxygen, superoxide anion radical, hydroperoxide, hydroxyl radical, and the lipid peroxide radical. Due to the brain's high utilization of oxygen; not adequate antioxidant defense and high amount of easily oxidizable, polyunsaturated fatty acids; and low concentration of serum antioxidants, it is especially sensitive to free radical injury [1]. Perinatal hypoxia occurs with an incidence of 2-6/1,000 term births. The neurological consequences of perinatal hypoxia-ischemia may be visible in 50-75% of children such as cerebral palsy, epilepsy, developmental delay, and hyperactivity disorder [2]. Hypoxic-ischemic brain injury is caused by a cascade of molecular reactions and mechanisms concerning calcium influx, free radical formation, free iron accumulation, nitric oxide production, and apoptosis activation. It is hypothesized that due to excessive free radical formation, the production of neurotrophic factors may be diminished and directly influence neurogenesis. The late precursor cells (preoligodendrocytes) and immature oligodendrocytes that predominate in the developing periventricular white matter during the period of highest risk for white matter damage in humans (23-32 weeks of postconceptional age) are particularly susceptible to hypoxia-ischemia [2, 3]. The studies of injured preterm brain proved the involvement of the white matter and the cortical and subcortical grey matter manifested as focal and/or diffuse lesions. The periventricular leucomalacia (PVL) as a focal lesion is seen in about 5% of children; more commonly, the changes are diffuse and coexist with intraventricular and germinal matrix hemorrhages [2]. According to Andiman et al., in the diffuse PVL, widespread axonal injury is usually recognized [3]. Still, little is known about the exact time of hypoxemia needed for hypoxia to occur. Some studies focusing on specific markers of brain injury—S-100B calcium-binding protein, neuron-specific enolase (NSE), glial fibrillary acidic protein (GFAP), ubiquitin carboxyl-terminal hydrolase l1 (UCH-L1), or total tau protein—evidence that astrocytes and neurons have been damaged and the proteins are released into the blood through the increased permeability of the blood-brain barrier [2, 3]. Due to poor specificity of cytokines, only interleukins IL-6 and IL-16 were correlated with HIE severity. Some authors claim that IL-6 and IL-8 as well as vascular endothelial growth factor (VEGF) may help to predict adverse neurological outcomes. However, the biomarkers specific for perinatal hypoxia are still lacking [4, 5].

## 2. Melatonin Secretion in Fetus and in Neonatal Period

The rhythmic secretion of endogenous melatonin appears around the age of 2-3 months in term neonates [6, 7], while nocturnal levels of melatonin may normalize even 48 hours after birth [8]. Preterm neonates display a delayed secretion of melatonin, which persists after correction for gestational age up to 8 to 9 months of age [8]. Some studies suggest that preterm babies do not produce melatonin until at least 52 weeks postconception [9]. In fetus life, melatonin is coming from the mother through the placenta. In the absence of maternal melatonin, the appearance of circadian rhythms depends principally on neurological maturation and very little on the environment [10]. Thus, short-gestation infants may suffer from melatonin deficiency, while full-term neonates may show only transient melatonin deficit. On the contrary, according to Tordjman et al., no correlation was found between the gestational age and the concentration of melatonin [11].

After birth, the neurologic network concerning the brain circuitry of melatonin secretion is immature, although the structures like the suprachiasmatic nuclei and pineal gland are well functioning in the prenatal period [6]. Bagci et al. stated that melatonin concentration in newborns is higher after vaginal delivery than after Caesarean section [12]. Among the other factors that may influence melatonin secretion profile are intrauterine growth restriction, premature rupture of the membranes (>6 hours), and preeclampsia (Karube 2001).

Human colostrum during the first 4-5 days after birth contains colostral mononuclear cells capable of synthesizing melatonin (autocrine synthesis). In newborns and infants, melatonin crosses the placenta and melatonin in the gastrointestinal tract is of maternal origin. Usually, the concentration of melatonin within the gastrointestinal tract is 10-100 times higher than that in the blood [11]. Melatonin secretion in children can be measured not only in blood but also in urine and saliva [13]. However, this complex physiological process can be disturbed in some disorders, for example, in epilepsy or Angelman syndrome [14–16].

## 3. Pathophysiology of Perinatal Encephalopathy

Hypoxic-ischemic encephalopathy (HIE) comprises abnormalities of consciousness, muscle tone, and autonomic control. The connection between the stage of HIE (I-III) and clinical presentation was initially reflected by the Sarnat staging system (Table 1). The Sarnat scale was introduced in 1976 for grading infants with hypoxic-ischemic encephalopathy based on EEG and neurological examination [17]. Later on, in the scoring system created by Thompson, a child status after birth asphyxia may be referred to as mild HIE (1-10 points), moderate HIE (1114 points), or severe HIE (15-22 points) [18, 19]. Assigning a numeric score gives detailed information on the grade of intrapartum hypoxia (Table 2).

Animal models showed the cascade of events occurring after hypoxia-ischemia. The pathophysiology of brain dysfunction in HIE in term and near-term infants is well described. Acute hypoxic-ischemic event provokes acute response—it starts from a rapid cell death due to necrosis so it is called a primary phase (from hypoxia and energy depletion, overactivation of glutamate receptors, and increased fee radical formation) [2, 20–23].

Secondary energy failure (delayed or programmed cell death) takes place 6-24 hours after a hypoxic event and lasts for days. This phase manifests itself by secondary cytotoxic edema, cytokines' secretion, and mitochondrial failure leading to cell death. Mitochondrial impairment (disruption of membrane integrity, loss of membrane potential) can determine overproduction of reactive oxygen molecules, abnormal calcium homeostasis, and production of apoptotic proteins [2, 20–23].

Tertiary phase (weeks to years) means persistent inflammation, epigenetic changes, limited oligodendrocytes' maturation, impaired neurogenesis and axonal growth, and altered synaptogenesis. According to the experimental study on neonatal animals by Eklind et al. [24], inflammation relative to hypoxia is most harmful for the developing brain when it appears acutely or in the chronic phase (probably because of a possible process of sensitization), whereas within the intermediate interval, it may have the opposite, protecting, effect. Thus, both intensity of the HIE insult and the developmental stage are of importance. The newborn brain is also sensitive to iron-mediated damage.

Because accurate prediction of neurodevelopmental outcome in neonatal encephalopathy is important for clinical management and to evaluate neuroprotective therapies, the prognostic accuracy of cerebral magnetic resonance (MR) biomarkers in infants with neonatal encephalopathy is worth to be taken into account and evaluated. A very interesting insight into hypoxic process pathology gives ^31^P brain spectroscopy. As revealed, during the first days after a hypoxic event, phosphocreatine (PCr) and adenosine triphosphate (ATP) are observed to fall down while Pi (inorganic phosphate) increases [25–27]. Also, the low cerebral PCr/Pi ratio and the increased brain lactate are associated with a poor developmental outcome. According to Cheong et al. [25] and Thayyil et al. [27], the lactate/N-acetyl aspartate (Lac/NAA) ratio on thalamic proton MRS (magnetic resonance spectroscopy) is a marker of neurodevelopmental outcome—its high values within the first 3–4 days after birth are predictive of poorer neurodevelopmental outcomes at 12-18 months of life. Thayyil et al. compared the sensitivity and specificity of early (1-7 days of life) and late (7-30 days of life) MRI with brain spectroscopy [27]. The authors concluded that MR spectroscopy is more informative than brain MRI performed in any time during the neonatal period [25, 27].

Another attempt to elucidate the complexity of the hypoxic-ischemic event was performed by Denihan et al. [26] using the metabolomic analysis. The authors applied a cord blood metabolic profiling based on direct-infusion Fourier-transform ion cyclotron resonance mass spectrometry (FT-ICR/MS). The metabolites, like melatonin, leucine, kynurenine, and 3-hydroxydodecanoic acid, allowed for differentiation between the children with perinatal asphyxia with recovery and the children with perinatal asphyxia followed by hypoxic-ischemic encephalopathy. HIE itself was associated with some abnormalities in tryptophan and pyrimidine metabolism [26].

The extent and neuropathology of white matter lesion may depend on fetus gestational age and severity of the insult. Significantly affected cerebral white matter will interrupt afferent and efferent cortical connections. Recent pathological studies have reported also grey matter involvement in perinatal injury, like neuronal loss, gliosis, and reduction in the density of pyramidal neurons (within layer 5) [3]. Such lesion can be observed in at least one-third of PVL cases in the basal ganglia and dentate cerebellar nuclei [28] and thalamus [29]. Cortical neuronal damage is either seen as a direct consequence of axonal and or somal destruction or subsequent to neuronal deafferentation [28–32].

Many MRI studies demonstrate diminished volumes of the thalamus and basal ganglia, hippocampus, and cerebellum [30, 31, 33, 34]. In term infants, neuronal injury was thought to predominate over injury to the white matter. However, recent assessment of the brains of term infants suggests that the same range of grey and white matter injury observed in preterm brains is sustained in term infants [35].

Probably, the genes involved in coagulation, inflammatory, and vascular pathways interacting with environmental facts may influence both the incidence and severity of cerebral injury. Polymorphisms in the Factor V Leiden gene are associated with the atypical timing of IVH, suggesting an as-yet-unknown environmental trigger [32]. The methylenetetrahydrofolate reductase (MTHFR) variants render neonates more vulnerable to cerebral injury in the presence of perinatal hypoxia [2, 32]. Probably, the MTHFR 677C>T polymorphism and low 5 min Apgar score additively increase the risk of IVH [2, 32]. Also, mutations in collagen 4A1, a major structural protein of the developing cerebral vasculature are seen.

Some of the children may experience fetal inflammatory response syndrome (FIRS) in the course of preterm delivery or premature and preterm rupture of the membranes (PROM). FIRS is a kind of immune system activation caused by IL-6 increased concentration [32]. It is still a matter of debate whether FIRS influences the response to HIE and whether HIE can induce FIRS and immune system activation [2, 32].

Originally defined in fetuses who experienced preterm labor and preterm premature rupture of the membranes (PROM), FIRS is a unique condition characterized by the systemic activation of the fetal innate immune system and by an elevation in fetal plasma IL-6 concentrations.

## 4. Perinatal Hypoxia Treatment Strategies

One of the well-known neuroprotective strategies in hypoxic-ischemic encephalopathy is moderate hypothermia (33-35 degrees Celsius) started within the first 6 hours after birth. Such therapeutic hypothermia is becoming a standard care for moderate to severe neonatal encephalopathy (NICE 2010) confirmed by a big clinical study (TOBY) [36, 37]. Seventy-two hours of hypothermia induced by head cooling [38] or systemic cooling [36, 39] within about 6 hours of birth was associated with a consistent reduction in death and neurological impairment at 18 months of age. Probably, the connection of hypothermia and pharmacological neuroprotection may be more effective. The future therapies are focused on prevention of brain damage and enhancement of endogenous brain repair mechanisms. There are many propositions of adjuvant therapy to supplement therapeutic hypothermia. New approaches to neuroprotection address different mechanisms. The proposed pharmacological action may use anti-inflammatory actions (erythropoietin (the BRITE study), melatonin, and xenon), antiapoptotic actions (inhibition of nuclear factor kappa B- and c-jun N-terminal kinase), and stimulation of neurotrophic properties: erythropoietin and brain-derived neurotrophic factor (BDNF) such as vascular endothelial growth factor (VEGF) and granulocyte colony-stimulating factor (GCSF) [40–43]. Among other antioxidants are allopurinol (ALBINO trial), topiramate, neuronal nitric oxide synthase (nNOS), inhibitors, pluronic copolymers, and cannabinoids [40–43]. Probably, stem cell therapy may offer stimulation of endogenous neural cells [40–43].

## 5. Melatonin: Its Possibilities and Pharmacokinetics in Preterm and Term Newborns

Recently, the protective possibilities of melatonin and its antioxidant actions have been studied in many neurodegenerative disorders like Alzheimer's disease, Huntington's disease, Parkinsonism, stroke, and ischemic brain injuries. The action of melatonin is mediated by two main receptors, MLT1 and MLT2, and an orphan receptor. The MLT1 receptor is mainly expressed in the suprachiasmatic nuclei, cerebellum, hippocampus, and pars tuberalis of the pituitary [1, 8, 9, 11]. The MLT2 receptor dominates in the retina but is also found in the suprachiasmatic nuclei, hippocampus, and cerebellum [1, 8, 9, 11]. Melatonin may serve as a potential therapeutic free radical scavenger and broad-spectrum antioxidant [1, 20–23].

Melatonin has neuroprotective properties against hypoxic-ischemic brain injury in animal models (Table 3).

The melatonin actions in the central nervous system lead to the following [8, 11, 44–46]:

- Increasing the number of neurons in melatonin-treated animals in the CA1, CA2–CA3 areas, and dentate gyrus of the hippocampus and parietal cortex
- Reduction of the expression of the glial fibrillary acidic protein
- Regulation of the expression of myelin basic protein and oligodendrocytes' function (regulation of myelination process)

Melatonin supplementation does not suppress the endogenous secretion of melatonin but is known to aid the establishment of appropriate circadian rhythms. After intravenous or oral administration, melatonin is metabolized, mainly in the liver and secondarily in the kidney [11]. While the pharmacokinetic profile of melatonin has been clearly documented in adults [47], in infants, the dosage of melatonin and the frequency of its administration may differ than those in adults. The exact dosage of this metabolite needed for neuroprotection is still unknown. In global or focal hypoxia-ischemia models, melatonin doses vary from 1 mg/kg to 50 mg/kg [46]. However, in the case of the preterm infants, the dosages cannot be extrapolated from the adult studies due to the infants' immature liver metabolism and poor renal excretion influencing the pharmacokinetic profile [9]. Merchant et al. [9] used in their clinical study melatonin concentrations closest to physiological adult concentrations and observed that compared with adults and older children, in preterm infants, the melatonin elimination half-life and the clearance were prolonged and the volume of distribution was decreased. However, as revealed from the animal and human data, the neuroprotective action of melatonin occurs at the doses much higher than the physiological ones. Such conditions were applied by Carloni et al. [48] in their study on the pharmacokinetics of melatonin after intragastric administration in preterm infants. Their main aim was to investigate the melatonin pharmacokinetics in human newborns at comparable doses. Thus, the authors used three different doses of melatonin: 0.1, 0.5, and 5 mg/kg, and extrapolated them according to the allometric evaluations [49] and the safety demonstrated by melatonin in clinical studies involving neonates with respiratory distress syndrome, bronchopulmonary dysplasia, and sepsis [50–53]. In such a way, they were able to cope with the main limitation for the use of melatonin in the neonatal clinical setting—the paucity of pharmacokinetic data with different delivery methods and dosages; however, their studied groups were also small as in Merchant et al. [9]. A different pharmacokinetic profile in premature newborns as compared to that of adults [47] and that of the experimental animals was confirmed in this study. Because melatonin is proposed as an adjunctive therapy to hypothermia for perinatal ischemic and inflammatory brain injuries [54], data from this study can be helpful to prescribe proper dosage and frequency of administration for this particular population.

Not only the adjustment of dose but also the choice of a proper timing to administer melatonin is of fundamental importance in the case of preterm and term infants (hours or days before or after hypoxia). During the perinatal and neonatal period, children are more exposed to oxidative stress as a consequence of the hyperoxic challenge due to the transition from the hypoxic intrauterine environment to extrauterine life and because of their reduced antioxidant defense mechanisms and increased susceptibility to highly toxic hydroxyl radical species produced in such processes as inflammation, hyperoxia, hypoxia, ischemia-reperfusion, neutrophil and macrophage activation, and glutamate and free iron release. The mitochondrial dysfunction, oxidative stress, inflammation, and cell death processes caused by an inadequate supply of ATP are involved in hypoxic-ischemic brain injury [1, 32, 55]. Accumulating evidence indicates that oxidative stress is implicated in the pathogenesis of many neurological diseases, such as intraventricular hemorrhage, hypoxic-ischemic encephalopathy, and epilepsy [55]. Thus, it is possible even to talk about “oxygen radical disease of the newborn” or “oxygen radical diseases of neonatology” [56, 57]. The preventive and therapeutic strategies have focused on avoiding the reactive oxygen species as well as using antioxidants. The peculiar perinatal susceptibility to oxidative stress indicates that prophylactic use of antioxidants as melatonin could help to prevent or at least reduce oxidative stress-related diseases in newborns [58]. However, more studies are needed to confirm the beneficial effects of melatonin in oxidative stress in the perinatal period. At the moment, there are several mainly animal studies showing the usefulness of melatonin in such applications.

## 6. Animal Models of Melatonin Protective Functions in Hypoxic-Ischemic Brain

The protective functions of melatonin against excitotoxicity, oxidative mitochondrial damage, inflammatory reaction, and cell death in the white matter have been studied for years in various animal models [59], but the details of the hypoxic-ischemic injury processes as well the of the mechanism(s) by which melatonin is neuroprotective still remain a subject of active investigation. Resent animal studies by Lin et al. [60] confirm that melatonin reduces intracerebral cellular inflammatory response and protects neurons against ischemic injury by reducing the oxidative stress, lipid peroxidation, and radical oxygen species generation. New possibilities open with the application of the spectroscopic techniques. Proton magnetic resonance spectroscopy was applied to study the effect of melatonin on neuropathology in response to perinatal asphyxia in newborn lambs [61]. The lactate : N-acetyl aspartate ratio was found to be much higher in asphyxia lambs compared with the controls, and melatonin prevented its rise. The authors concluded that melatonin administered soon after birth may improve the outcome in infants affected by asphyxia.

Carloni et al. have found out positive effects of melatonin administration before and after hypoxia in immature rats [59]. In 2017, they showed in a neonatal rat brain increased expression and activity of SIRT1 (silent information regulator 1), reduced expression and acetylation of p53, and increased autophagy activation [48]. In the experimental study performed by Yawno et al. in a preterm fetal sheep, an increased oligodendrocyte cell number within the periventricular white matter and improved CNPase+ myelin within the subcortical white matter were found [62].

Robertson et al. in the piglet study demonstrated that combination of melatonin (5 mg/kg) and hypothermia (33.5°C) is more effective than any of these forms of the treatment alone [46]. Signorini et al. [63] investigated the possible effect of melatonin supplementation in a rat pump model of hypoxic-ischemic encephalopathy and proved that hypoxia may induce formation of desferrioxamine-chelatable free irons in the cerebral cortex and, in consequence, increase oxidative stress. The latter, however, can be prevented by melatonin administrated before starting the ischemic procedure.

According to Alonso-Alconada et al., the treatment with melatonin at 15 mg/kg after neonatal hypoxia-ischemia led to reducing cell death, white matter demyelination, and reactive astrogliosis in rat pumps [64, 65].

Melatonin was shown to protect against ischemia-/reperfusion-induced oxidative damage to mitochondria in the fetal rat brain. According to an experimental study by Watanabe et al. performed in pregnant rats, melatonin may reverse the hypoxia-/reperfusion-induced changes measured as respiratory control index (RCI) and thiobarbituric acid-related substances (TBARS) [66]. Hutton et al. showed lower levels of fractin and caspase-3 activation after melatonin administration in a spiny mouse [67]. Another, already mentioned, experimental study conducted by Robertson et al. (piglet model of hypoxia-ischemia) discovered a positive effect on repeated melatonin infusion on ^1^H MRS biomarkers (lactate/N-acetyl aspartate, lactate/creatinine) [46].

There are also some biochemical studies carried out in animal models and in vitro systems focused on the chelating properties of melatonin. Romero et al. summarize them in their review on the current state of knowledge related to the role of metals in the generation of free radical species and tissue injury and on the protective role of melatonin against metal-induced toxicity [68]. However, also in this area, further studies are necessary, as the authors claim, to confirm the usefulness of melatonin against metal-induced toxicity.

## 7. Melatonin and the Clinical Trials

Data from the mentioned studies can be used to guide therapeutic clinical trials of melatonin in preterm infants. Unfortunately, we currently lack clinical trials with a sufficient sample size to confirm the benefit of melatonin treatment in neonates with hypoxic-ischemic encephalopathy in hypothermia.

Table 4 presents the main results of our review of the already performed studies or trials.

We took into account 8 reports; however, there is only one project concerning neonates with hypoxic-ischemic encephalopathy with melatonin adjuvant treatment which is in line with our main subject. The data included in Table 4 show that the dosages, diagnosis, and observed clinical factors differ markedly in various approaches, thus, making the comparisons of the results difficult or impossible.

Aly et al. (Table 4) performed a prospective trial of 30 HIE newborns randomized in the hypothermia alone and hypothermia with melatonin group on the background of the healthy newborns [69]. All infants were studied with repeated EEG and brain MRI. In all babies, superoxide dismutase (SOD) and nitric oxide (NO) were measured. Melatonin administration to neonates with hypoxic-ischemic encephalopathy preserved the serum SOD concentration, reduced the production of NO, and ameliorated brain injury. These examinations showed also increased melatonin and decreased NO in the hypothermia-melatonin group [69].

Gitto et al. (Table 4) carried out a series of studies in preterm infants with melatonin adjuvant usage compared with conventional treatment [50–52]. In these works, the authors involved the cases with respiratory distress syndrome and bronchopulmonary dysplasia and compared the concentrations of the inflammatory mediators IL-6, IL-8, TNF*α*, and nitrite/nitrate.

There are additional two important reports concerning the pharmacokinetic profile of melatonin in preterm infants (Table 4)—the data collected in these trials can be used to guide therapeutic clinical trials of melatonin in preterm infants in the future. In that of Merchant et al. [9], the group size is small (only 18 preterm babies); however, the authors were able to show that the dosage of melatonin for use in preterm infants cannot be extrapolated from adult studies. Because of the postulated unpredicted bioavailability of oral melatonin in this trial, melatonin was administered intravenously to 18 preterm infants (less than 31 weeks of gestation, less than 7 days old) in doses ranging from 0.04 to 0.6 *μ*g kg^−1^ over 0.5–6 h in order to attain the physiological adult concentrations. The results of the trial led by Carloni et al. [70] point out the possibility to obtain and maintain neuroprotective concentrations of melatonin in the blood using a single oral administration of indolamine repeated every 12/24 h. Unfortunately, also in this study, the sizes of the studied groups of children were small (3 × 5 newborns); however, the methodology of the doses scaling between various approaches may be helpful in the future.

Another challenge might be the possibility to administer melatonin antenatally in order to prevent or reduce brain hypoxic insult in preterm babies as shown in ClinicalTrials.gov Identifier NCT01340417 [8].

## 8. Genetic Studies

A very interesting study on melatonin influence on neural stem cell (NSC) functioning was conducted by Fu et al. [71]. Under hypoxic conditions, the authors showed that melatonin treatment may induce upregulation of Mash1, Neurog1, and NeuroD2 in the differentiated NSCs compared with untreated cells in hypoxia [71].

Denihan et al. worked on metabolomic biomarkers of umbilical cord blood after hypoxic injury [26]. They checked different parameters using DI FT-ICR (Fourier-transform ion cyclotron resonance spectrometry). Some metabolites allowed for differentiation between children with perinatal asphyxia with recovery and children with perinatal asphyxia followed by hypoxic-ischemic encephalopathy like melatonin, leucine, kynurenine, and 3-hydroxydodecanoic acid. HIE itself was associated with abnormalities in tryptophan and pyrimidine metabolism [26].

Children after hypoxia-ischemia brain injury often develop circadian rhythm disorders. Yang et al. documented that mRNA and protein expressions of pineal arylalkylamine N-acetyltransferase (AANAT) and melatonin are impaired after hypoxic damage [72]. They postulated that miR-325-3p (microRNA) may play a role of a potential downregulator of AANAT-rate-limiting enzyme for melatonin synthesis [72].

Another interesting point is that hypoxic-ischemic-induced inflammation in preterm animals may be partially responsible for blood-brain barrier disruption and white matter injury [73–75]. Hu et al. in an experimental study on hypoxic-ischemic brain injury in rat pups showed that melatonin treatment may restore decreased pericyte markers: PDGFR*β* and desmin [75].

## 9. Conclusion

As stated above, the studies that focus on the neuroprotective potential of melatonin and its possibility of treatment after perinatal asphyxia in humans are scarce. The melatonin advantages—its efficacy, low toxicity, and ability to readily cross through the blood-brain barrier—make it a promising molecule. A very interesting thing is the difference between the half-life of melatonin in preterm neonates (15 hours) and adults (45-60 minutes). Hypoxic-ischemic insult leads to devastating neurological consequences—that is why there is a quest for other therapies complementary to the therapeutic hypothermia (in term infants). Such novel approaches are still necessary, but they require to be validated in randomized trials. Probably, the synergic strategies coupled with melatonin treatment may be promising options improving antioxidant action.

## Figures and Tables

**Table 1:** Sarnat scale [17].

	Stage 1	Stage 2	Stage 3
Mental state	Hyperalert	Lethargic or obtunded	Stuporous
Cranial nerves	Weak suck	Weak or absent	Absent
Motor	Normal	Mild hypotonia	Severe hypotonia
Reflexes	Mildly brisk	Brisk	Suppression
Primitive reflexes	Normal	Suppressed	Suppression
Autonomic reflexes	Sympathetic activation	Parasympathetic activation	Both systems suppressed
Seizures	None	Common	Uncommon
EEG	Normal	First day low voltage, the burst suppression pattern and multifocal electrographic seizures	Deep periodic EEG with burst suppression pattern
Duration	Less than 24 h	2-14 days	Hours to weeks
Prognosis	No sequelae	Good prognosis if recovery is within days	Microcephaly, psychomotor development retardation, seizures, cerebral palsy

**Table 2:** Thompson scale [19].

Signs	Score			
	0	1	2	3
Tone	Normal	Hyper	Hypo	Flaccid
Level of consciousness	Normal	Hyperalert, stare	Lethargic	Comatose
Fits	None	<3/day	>2/day	
Posture	Normal	Fisting, cycling	Strong distal flexion	Decerebrate
Moro	Normal	Partial	Absent	
Grasp	Normal	Poor	Absent	
Suck	Normal	Poor	Absent+/- bites	
Respiration	Normal	Hyperventilation	Brief apnea	IPPV (apnea)
Respiration	Normal	Full, not tense	Tense	

**Table 3:** Proposed antioxidant actions of melatonin [1, 9, 44].

Melatonin	Scavenging of free radicals	Hydroxyl radicals, hydrogen peroxide, singlet oxygen
	Upregulating antioxidant pathways	Superoxide dismutase, glutathione, catalase, glutathione peroxidase, glutathione reductase
	Reduction of lipid peroxidation	Excessive production of malondialdehyde
	Stimulation of mitochondrial biogenesis and promotion of the electron transport chain	Avoided mitochondrial permeability transition pore opening, decreased the number of TUNEL^∗^-positive cells/DNA breaks
	Readily crossing both the placental and blood-brain barriers	
	Antiapoptotic action	Prevention of cytochrome *c* release, reduced or blocked caspase-1 and caspase-3 activation, increased the expression of anti-apoptotic proteins Bcl-2 and Bcl-xL, diminished Bad and Bax pro-apoptotic proteins, inhibited poly-ADP-ribose-polymerase cleavage

^∗^TUNEL: terminal deoxynucleotidyl transferase dUTP nick end labeling

**Table 4:** Outcome definition details of the studies included in the review.

Article title	Source	Population	Melatonin groups (*n*)	Nonmelatonin groups (*n*)	Melatonin dose, route	Effects observed and investigated clinical factors	Result of melatonin adjuvant treatment
“Melatonin Use for Neuroprotection in Perinatal Asphyxia: a Randomized Controlled Pilot Study”	Aly H et al. J Perinatol 2015; 35: 186-191	Neonates with hypoxic-ischemicencephalopathy (HIE)	15 HIE newborns	15 HIE newborns; 15 healthy	Total of 50 mg/kg as 5 daily enteral doses	Serum melatonin, plasma superoxide dismutase, serum nitric oxide, electroencephalography, magnetic resonance imaging, neurologic evaluations	Positive impact on the studied clinical factors

“Increased Levels of Malondialdehyde and Nitrite/Nitrate in the Blood of Asphyxiated Newborns: Reduction by Melatonin”	Fulia et al. J. Pineal Res. 2001; 31 : 343-349	Asphyxiated newborns	10 asphyxiated newborns	10 asphyxiated newborns	Total of 80 mg as 8 oral doses	Serum malondialdehyde and nitriate/nitrate concentrations	Positive impact on the studied clinical factors

“Correlation among Cytokines, Bronchopulmonary Dysplasia and Modality of Ventilation in Preterm Newborns: Improvement with Melatonin Treatment”	Gitto et al. J. Pineal Res. 2005; 39:287-293	Preterm infants with respiratory distress syndrome	55 preterm infants	55 preterm infants	Total of 100 mg/kg as 10 infusions	Interleukin measurements (IL-6, IL-8, and TNF*α*)	Positive impact on the studied clinical factors

“Oxidative and Inflammatory Parameters in Respiratory Distress Syndrome of Preterm Newborns: Beneficial Effects of Melatonin”	Gitto et al. Amer J Perinatol 2004; 21 (4): 209-2016	Preterm infants with respiratory distress syndrome	40 preterm infants	34 preterm infants	Total of 100 mg/kg as 10 infusions	Inflammatory mediators (IL-6, IL-8, TNF*α*, and nitrite/nitrate) measurements	Positive impact on the studied clinical factors

“Early Indicators of Chronic Lung Disease in Preterm Infants with Respiratory Distress Syndrome and Their Inhibition by Melatonin”	Gitto et al. J. Pineal Res. 2004; 36 (4): 250-5	Preterm infants with respiratory distress syndrome	60 preterm infants	60 preterm infants	Total of 100 mg/kg as 10 infusions	Inflammatory mediators (IL-6, IL-8, TNF*α*, and nitrite/nitrate) measurements	Positive impact on the studied clinical factors

“Pharmacokinetics of Melatonin in Preterm Infants”	Merchant et al. Br J Clin Pharmacol 2013; 76: 725733.	Preterm infants	18 preterm infants		Total of 0.04-0.6 *μ*g/kg over 0.5-6 h as infusion	Pharmacokinetic profiles	Positive impact on the studied clinical factors

“Melatonin as a Novel Neuroprotectant in Preterm Infants—a Double Blinded Randomised Controlled Trial (MINT Study)”	Merchant et al. Arch Dis Child 2014: 99 (Suppl 2):A1-A620	Preterm infants	30 preterm infants	28 preterm infants	Total of 0.2 *μ*g/kg over 2 h as infusion	Fractional anisotropy on magnetic resonance imaging	No effect on the studied clinical factors has been observed

“Melatonin Pharmacokinetics Following Oral Administration in Preterm Neonates”	Carloni et al. Molecules 2017; 22, 2115	Preterm infants	5 preterm infants with low dose, 5 preterm infants with medium dose, 5 preterm infants with high dose		Total of 0.5 mg/kg or 3 mg/kg or 15 mg/kg as 1 or 3 intragastric boluses	Pharmacokinetic profiles	Positive impact on the studied clinical factors
